# Single Nucleotide Polymorphism (rs4932178) in the P1 Promoter of *FURIN* Is Not Prognostic to Colon Cancer

**DOI:** 10.1155/2015/321276

**Published:** 2015-06-07

**Authors:** Jeroen Declercq, Bart Jacobs, Bart Biesmans, Arnaud Roth, Dirk Klingbiel, Sabine Tejpar, John W. Creemers

**Affiliations:** ^1^Laboratory for Biochemical Neuroendocrinology, Department of Human Genetics, KU Leuven, 3000 Leuven, Belgium; ^2^Digestive Oncology Unit, Department of Human Genetics, KU Leuven, 3000 Leuven, Belgium; ^3^Oncosurgery Unit, University Hospital of Geneva, 1204 Geneva, Switzerland; ^4^Swiss Group for Clinical Cancer Research (SAKK) Coordinating Center, 3008 Bern, Switzerland

## Abstract

High expression of the proprotein processing enzyme FURIN has been associated with tumor progression and metastasis. A SNP (rs4932178) in the promoter of *FURIN* has been reported to affect expression in liver, with the T allele resulting in higher expression than the C allele. In this study we have investigated the association of this SNP with prognostic and biological subgroups of colorectal cancer (CRC). In a panel of 1382 patients with CRC, this SNP had no impact on overall survival or on postoperative risk of relapse. This SNP also could not be linked with *FURIN* expression levels in CRC samples from the patients. Furthermore, we demonstrate in luciferase reporter experiments in the colon cancer cell lines Caco-2 and SW480 and in the hepatocellular carcinoma cell line Huh 7 that expression is not affected by the SNP. Since, FURIN inhibition in human colon cancer cell lines has previously been shown to repress tumor metastases, association between *FURIN* gene expression levels and postoperative relapse-free survival was also investigated. However, no association could be found. Altogether, we could not confirm an effect of the SNP on *FURIN* expression *in vitro* and no correlations could be found *in vivo* with *FURIN* expression or outcome.

## 1. Introduction

Colorectal cancer (CRC) ranks second to lung cancer in both incidence and mortality in developed countries [1]. The identification and validation of new therapeutic targets to combat this disease are therefore of the utmost importance. This goal is, however, complicated by the fact that CRC is a very heterogeneous disease, where clinicopathological seemingly similar tumors behave very different in terms of treatment response and patient survival. Therefore, a therapeutic strategy with a broad effect that is not restricted to a single pathway has a higher potential to be successful. The proprotein convertase Furin was shown to be involved in many cancer types. Genetic ablation of* Furin* in a mouse model for salivary gland tumors significantly delayed the tumor formation [2], while transgenic mice overexpressing* Furin* in the epidermis show enhanced skin cancer development [3]. Furin downregulation in colon carcinoma cell lines inhibited the processing of IGF1R and reduced liver metastases after injection into the portal vein of mice [4]. Targeting Furin might be a potential therapeutic strategy affecting multiple pathways simultaneously. Furthermore, recently the first specific FURIN inhibitors were generated [5] and now need to be validated in therapeutic applications.

Furin is an endoprotease that cleaves carboxyterminal of specific basic amino acid motifs and thus activates a variety of precursor proteins [6, 7]. These precursor proteins include growth factors and differentiation factors, receptors, adhesion molecules, and enzymes like metalloproteases (MMPs). These factors play important roles at different stages of tumor development, progression, vascularization, and metastasis. Therefore, it is not surprising that* FURIN* is highly expressed in various tumor cell lines and human primary tumors [8]. Furthermore, it has been shown that inhibition, knockdown, and genetic ablation of FURIN reduce tumorigenesis in various human cancer cells [4]. For example, FURIN inhibition in squamous cell carcinoma cell lines resulted in a decreased proliferation, reduced the anchorage-independent growth in soft agar assays, and inhibited the* in vivo *tumorigenicity and invasion in nude mice [9]. In contrast, FURIN overexpression in these cell lines resulted in the opposite phenotype and increased the proliferation and invasiveness [10]. This is also the case in mice. Transgenic mice, overexpressing Furin, display enhanced skin tumor formation [3]. Likewise, we previously demonstrated that genetic ablation of* Furin* in the salivary glands inhibited the development and progression of* PLAG1*-induced pleomorphic adenomas of the salivary glands [2]. Inactivation of only a single* Furin *allele already resulted in a significantly delayed onset of tumorigenesis. This suggests that therapeutic benefit can be achieved even with partial inhibition.* PLAG1*-induced tumors utilize the Igf1-receptor (Igf1r) pathway, which is relevant not only in salivary gland tumors but also in several other types of cancer including CRCs [11–15]. Studies in CRC cell lines suggest that FURIN inhibition can repress the metastatic potential [4]. This suppressive effect is mediated via the inhibition of IGF1R processing. As a result, IGF1-induced AKT phosphorylation, an important step in colon carcinoma metastasis, is lost.

The expression of* FURIN* is regulated by three different promoters, resulting in three distinct* FURIN* mRNA isoforms which differ only in their 5′-untranslated regions [16]. Promoter P1 contains a TATA box, is transactivated by C/EBP*β*, the transcription factor SP1 [16], hypoxia-inducible factor-1 [17], SMAD2/SMAD4 [18], and Gata-1 [19], among others. The other two promoters (P1A and P1B) lack TATA or CAAT boxes and contain architectural features of housekeeping promoters.

A SNP (rs4932178) in the P1 promoter of the* FURIN* gene has been reported to affect the expression levels of FURIN about 3-fold (T allele higher than the C allele) in HepG2 and HuH7 cell lines [20]. Individuals carrying T allele were more likely to become persistently infected with hepatitis B virus infection. This virus requires FURIN for HBeAg maturation and hence immune response evasion. This SNP has also been analyzed in a group of 299 patients with CRC [21]. In this study, the carriers of the CT genotype of FURIN C-229T had a worse relapse-free and overall survival than the carriers of the CC genotype. However, no effect on survival was observed for the rare TT genotype, diminishing the value of this finding. Therefore, those studies should be validated in larger, independent studies. Whether or not* FURIN* expression (independently of the SNP) can be linked with a worse survival probability of patients with CRC has not been investigated yet.

In this study, the postoperative relapse-free survival and the survival time of large panel of patients with CRC have been investigated in correlation with SNP C-229T and expression of* FURIN*. Furthermore, the effect of this SNP on expression of* FURIN* in CRC cell lines was determined.

## 2. Materials and Methods

### 2.1. Patients Characteristics

The trial was a nonblinded multicenter randomized phase III study conducted within the Pan-European Trial in Adjuvant Colon Cancer network as described previously [22]. A total of 1382 patients with stages II to III adenocarcinoma of the colon were selected. All patients were between 18 and 75 years old. The aim of this trial was to assess whether the addition of irinotecan to de Gramont infusional fluorouracil/leucovorin would improve disease-free survival (DFS) in patients with stage III colon cancer. Trial design and the identification of several prognostic markers based on the trial data were reported previously [23–28]. The trial was conducted according to the Declaration of Helsinki and its conduction was monitored by a steering committee and an independent data monitoring committee.

### 2.2. SNP Analysis

Multiplex PCR was performed in a 5 *μ*L volume containing MegaMix Gold (Cambio), 5–10 ng of genomic DNA, and 100 nM of each PCR primer. Thermocycling was performed at 95°C for 15 min, followed by 45 cycles of 94°C for 20 s, 56°C for 30 s, and 72°C for 60 s, followed by a final extension of 72°C for 3 min. Unincorporated dNTPs were deactivated using 0.3 units of shrimp alkaline phosphatase (Clontech Laboratories, Inc., Mountain View, USA) at 37° for 40 min and primer extension was carried out using 7–14 *μ*M of each primer extension probe (depending on the mass), 1 unit of iPLEX termination mix, and 1 unit of iPLEX enzyme.

Reactions were cycled at 94°C for 30 s, followed by 44 cycles of 94°C for 5 s, 5 times (52°C for 5 s and 80°C for 5 s). After the addition of a cation exchange resin (Sequenom Inc.) to remove residual salt from the reactions, 20 *μ*L of water was added and the extension product was spotted onto a matrix pad (3-hydroxypicoloinic acid) of a SpectroCHIP (Sequenom Inc.). After analyzing the SpectroCHIPs using a MALDI-TOF mass spectrometer, spectra were processed by the SpectroREADER software (Sequenom Inc.) and transferred to the MassARRAY Typer 4 Analyzer (Sequenom Inc.) for further analysis. Genotyping for every sample was performed using the default settings of the MassARRAY Typer 4 Analyzer. Genotyping calls were generated and were validated by manual review of the raw mass spectra.

### 2.3. Microarray Analysis

Microarray analysis was performed on CRC tumor samples of 688 patients as described previously [29]. In brief, RNA of sufficient quantity and quality was extracted from the tumor samples, and gene expressions were measured on the ALMAC colorectal cancer DSA platform (Craigavon, Northern Ireland) with a customized Affymetrix chip with 61,528 probe sets mapping to 15,920 unique Entrez Gene IDs. Three different probe sets were used to analyze the expression of* FURIN*: CB852900_s, NM_002569, and NM_002569_x.

### 2.4. Site Directed Mutagenesis

pGL2-P1-SacI construct, here referred to as pGL2-P1C which contains a DNA fragment, starting at the Pst1 site in exon 1 and extending to the Sac1 site 2,6 kb upstream, in the luciferase construct pGL2 has previously been described [16]. This construct contains part of the human P1 promoter of* FURIN* containing the C allele of the SNP −229C/T (rs4932178). The QuickChange site directed mutagenesis kit (Stratagene) was used to mutate this C into a T according to the instructions of the manufacturer, using the primers: 5′-GGTAAGTGCAGACTCACCCCAATAAATGAGG-3′ and 5′-CCTCATTTATTGGGGTGAGTCTGCACTTACC-3′. The resulting construct, referred to as pGl2-P1T, was sequenced to confirm the mutation.

### 2.5. Luciferase Assay

500 ng of the plasmids pGL2-P1C and pGL2-P1T and pGL2-basic were transfected in HuH7, Caco-2, and SW480 cells using FuGENE 6 as a transfection reagent according to the manufacturer's protocol in 24-well plates. 50 ng/well of pRL-tk (Renilla luciferase expression construct, Promega) was used for normalization of the transfection efficiencies. Each construct was transfected at least three times in triplicate. 24 hours after transfection, cells were lysed and assayed for luciferase activity using the dual luciferase assay system (Promega) according to the instructions of the manufacturer.

### 2.6. Statistical Analysis

The observed allele frequencies were tested for Hardy-Weinberg equilibrium and the differences between the observed and the expected frequencies were tested for significance using the Chi-square test. Kaplan-Meier methods were used to estimate the survival probabilities (postoperative relapse-free survival and overall survival) and the log-rank test was used to assess differences between patients with the three different SNPs. The association between the expression of* FURIN *and overall survival was analyzed by Cox regression using continuous* FURIN* expression values. The hazard ratio (HR) and the 95% CI were determined with the CC allele as reference level.

The unpaired *t*-test was used to analyze the data for the luciferase experiments. The association of the alleles with gene expression levels was done with the Kruskal-Wallis test. *P* values are two-sided, considered significant if <0.05 and not adjusted for multiple testing. Statistical analyses have been performed using R version 2.12.0 or later (http://www.r-project.org/).

## 3. Results

**Figure 1:**
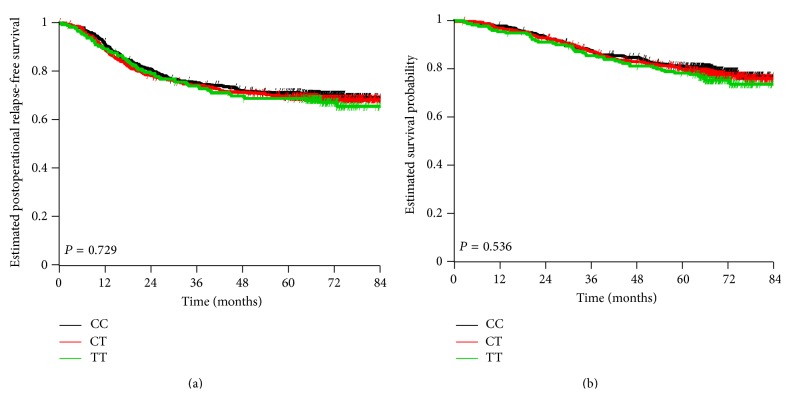
(a) Kaplan-Meier curve of the postoperational relapse-free survival of CRC according to the patients genotypes of the SNP rs4932178 in the* FURIN* promoter, C-229T (CC versus CT, *P* = 0.56; CC versus TT, *P* = 0.47). (b) Kaplan-Meier curve of the overall survival of CRC according to the patients genotypes of the SNP rs4932178 in the* FURIN* promoter, C-229T (CC versus CT, *P* = 0.57; CC versus TT, *P* = 0.27). The *P* values in the figure are from the global log-rank test, showing no evidence of a difference between the three groups.

SNP rs4932178 was successfully determined for 1366 of the 1382 patients with stage II to stage III adenocarcinoma in the cohort (Table 1). The distribution frequencies of the genotypes CC, CT, and TT were 38.7% (529/1366), 47.6% (650/1366), and 13.7% (187/1366), respectively. No evidence of a violation of the Hardy-Weinberg equilibrium was found. Further, no statistically significant association was found between genotypes in clinical and molecular subgroups (Table 3). Among patients with the different SNPs in the* FURIN* promoter, no significant differences in the relapse-free survival (Figure 1(a)) and the overall survival (Figure 1(b)) could be observed. CT genotype carriers showed no differences in relapse-free survival compared to CC genotype carriers (HR: 1.07, CI: 0.86–1.32, *P* = 0.56) or in overall survival (HR: 1.08, CI: 0.83–1.39, *P* = 0.57). Likewise, TT genotype carriers showed no differences in relapse-free survival compared to CC genotype carriers (HR: 1.12, CI: 0.83–1.52, *P* = 0.47) or in overall survival (HR: 1.22, CI: 0.86–1.73, *P* = 0.27).

**Figure 2:**
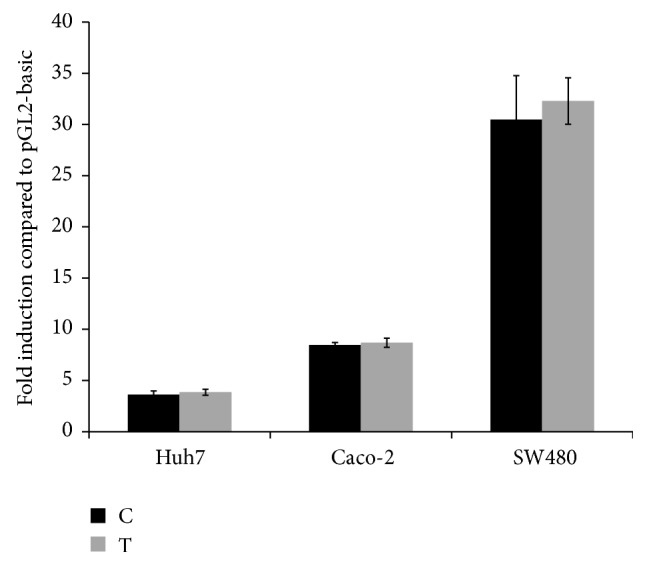
Luciferase activity of −229C and −229T alleles in the P1 promoter of the* Furin* gene in three different cell lines: Huh7, Caco-2, and SW480. Results are shown as fold induction compared to the luciferase activity in the pGL2-basic vector ± SEM of at least three independent experiments. No statistically significant differences could be observed using the Student's *t*-test; *P* = 0.66, *P* = 0.79, and *P* = 0.69, respectively.

SNP rs4932178 in the promoter of* FURIN* has been reported to influence* FURIN* expression in hepatocellular carcinoma cell lines [20]. To investigate whether this is also the case for colon cancer cell lines, the impact of the two alleles of the SNP on the transcription was analyzed in the hepatocellular carcinoma cell line Huh7 and in two colon cancer cell lines Caco-2 and SW480 by luciferase experiments. In contrast to the previous report, there were no significant differences in the luciferase activity using pGL2-P1T or pGL2-P1C constructs in Huh7, Caco-2, and SW480 cells (Figure 2). This suggests that the SNP has no impact on the* FURIN* expression in those cell lines.

**Figure 3:**
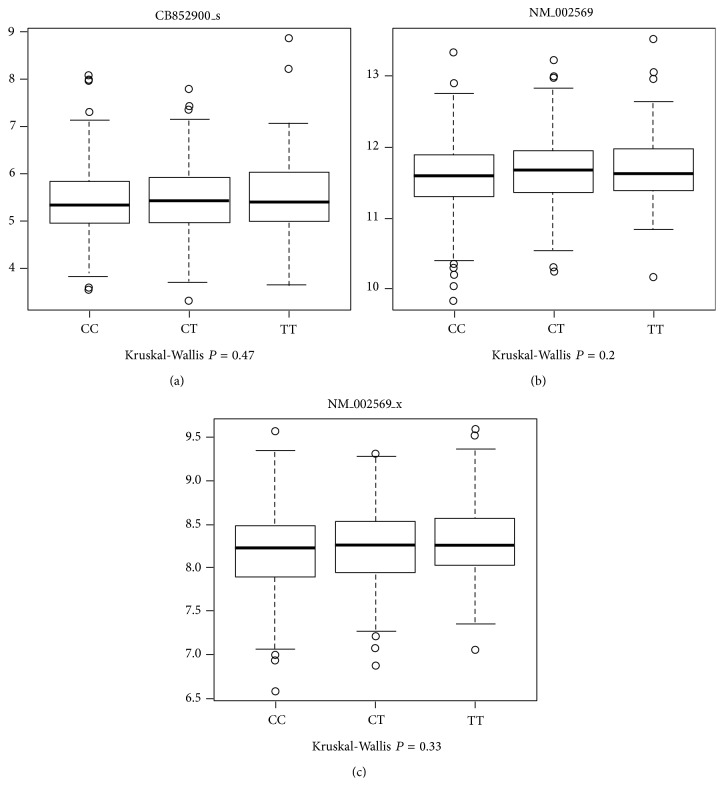
Boxplot of the gene expression levels of* FURIN *according to the patient's genotypes of the SNP rs4932178 in the* FURIN *promoter, C-229T, for three different probe sets: (a) CB852900_s, (b) NM_002569, and (c) NM_002569_x. No statistical significant differences could be observed using the Kruskal-Wallis test; *P* = 0.47, *P* = 0.2, and *P* = 0.33, respectively.

We subsequently analyzed whether or not the SNP in the* FURIN* promoter had an impact on* FURIN* expression in the subset of CRC tumor samples of 688 patients for which gene expression data were also available. Consistent with the results of the luciferase assay, there were no differences in the* FURIN* expression among patients with CC, CT, or TT genotypes of the SNP using three different probe sets (Figure 3).

It has been reported that FURIN inhibition in human colon cancer cell lines inhibits the metastatic potential of those cell lines [4]. Therefore, we have examined whether or not* FURIN* expression by itself is linked with relapse-free survival in this group of 688 patients with colon cancer. Using cox regression analysis with continuous* FURIN* expression values, no association could be found for* FURIN* expression and relapse-free survival for the three different probe sets (Table 2) (CB852900_s HR: 1.02, CI: 0.87–1.20, *P* = 0.81; NM_002569 HR: 0.98, CI: 0.77–1.25, *P* = 0.90; NM_002569_x HR: 0.94, CI: 0.72–1.22, *P* = 0.65). Thus, when the* FURIN* expression increases, we found no increased risk for tumor relapse after the surgery.

## 4. Discussion

In this paper we show that SNP (C-229T) in the* FURIN* promoter is not prognostic to CRC. The distribution pattern of the CC, CT, and TT genotype carriers was determined in 1366 patients with CRC. This pattern (CC: 38.7%, 529/1366; CT: 47.6%, 650/1366; TT: 13.7%, 187/1366) was similar to that of a previous report performed on Swedish patients with CRC (CC: 34.8%, 104/299; CT: 51.8%, 155/299; and TT: 13.4%, 40/299) (*P* = 0.38) [21]. However, the distribution of the SNP is significantly different in healthy adults from southern China (CC: 67.9%, 57/84; CT: 26.2%, 22/84; TT: 5.9%, 5/84) (*P* < 0.0001) and in Chinese HBV-infected patients (CC: 61.1%, 374/612; CT: 30.1%, 184/612; TT: 8.8%, 54/612) (*P* < 0.0001) [20]. Thus, there are differences in the distribution of the SNP depending on the ethnic background.

**Figure 4:**
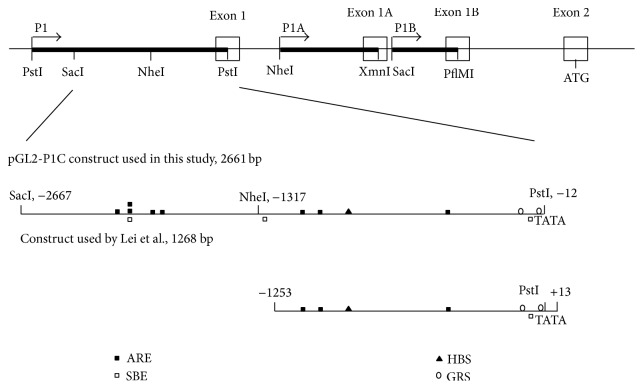
Schematic representation of the three different* Furin* promoters (P1, P1A, and P1B), the pGL2-P1C construct used in this study, and the construct used by Lei and coworkers. The positions are determined from the mRNA sequence, GenBank accession number: NM_002569. Elements important for* FURIN *expression are indicated. ARE = activin responsive element; SBE = smad binding elements; HBS = HIF-1 consensus binding sequence; GRS = Gata-1 recognition sequence.

Previously, it has been reported that CT genotype carriers of a SNP in the* FURIN* promoter showed a worse survival than homozygous CC genotype carriers [21]. However, this was not the case for the rare TT genotype carriers. Therefore, these results were inconclusive and requested a validation in a larger group of patients in an independent study. In our independent study in 1382 patients with CRC, no differences in overall or relapse-free survival were observed depending on this SNP. This suggests that the differences in overall survival observed for the CT genotype carriers in the previous report were indeed only a coincidence. Furthermore, the expression of* FURIN* in the CRC was also similar in patients with different SNPs in the* FURIN* promoter. Consistent with this observation, no differences in luciferase activity could be observed in luciferase reporter assays in colon cancer cell lines after transfection with a construct containing the C or the T allele of the SNP. This was also not the case in the hepatocellular carcinoma cell line Huh7. This result is in contrast to a previous report, where a 3-fold increase in the transcriptional activity was observed in Huh7 cells after transfection with a reporter construct containing the T allele of the SNP compared to one containing the C allele [20]. The reason why this is the case is not completely clear but might be due to the different region of the P1 promoter that was used for the luciferase reporter assay. The construct used in this study contains 2661 bp of the P1 promoter while the construct used by Lei and coworkers contained only 1268 bp of the P1 promoter (Figure 4) [16, 20]. Since* FURIN *expression in the HBV-infected patients has not been investigated directly, it is unclear whether or not the increased expression* in vitro*, as observed by Wei and coworkers, is a reflection of the expression* in vivo*. Since our* in vitro* results are consistent with the* in vivo* expression data, it is tempting to speculate that the larger fragment of the P1 promoter contains additional elements relevant for* FURIN* expression. The most proximal region of the P1 promoter, which contains the TATA box and which is included in both constructs, contains most of the elements required for constitutive promoter function [16]. Nevertheless, it fails to respond significantly to TGF*β*1 stimulation [18]. The 809 bp region between positions −1317 and −508 carries most of the transcriptional activation of the* FURIN* P1 promoter by TGF*β*1. This can be explained by the presence of multiple putative activin responsive elements (ARE) and a Smad binding element (SBE) in this region. It should be noted that the SBE is only present in our construct but not in the construct used by Lei and coworkers. Likewise, several other putative ARE binding sites and SBE binding sites are located upstream of the −1253 position and are only included in the construct used in this study (Figure 4). The presence of those additional elements relevant for* FURIN* expression can thus explain the differences observed by Lei and coworkers. In any case, it is clear that differences in this SNP in the P1 promoter of* FURIN* do not affect* FURIN* expression in CRC and that this SNP has no predictive outcome in this tumor type. Likewise, also in human atherosclerotic plaques the SNP C-229T was not found to be associated with* FURIN* expression [30].

We have also studied whether or not* FURIN* expression by itself has a predictive outcome in colon cancer. Indeed, FURIN inhibition in human colorectal tumor cells repressed tumor metastases via inhibition of IGF1R processing in mouse models [4]. Therefore, we investigated the association between* FURIN* expression and relapse-free survival in patients with CRC but not stratified for IGF1R pathway activation. No association could be found. Although this might suggest that FURIN inhibition is of limited value for patients with CRC, it may also be that the expression of* FURIN* in all tumors is sufficiently high not to be limiting in the processing of substrates. It the latter case, it remains well possible that inhibition below a certain threshold will provide therapeutic benefit.

In contrast to patients with CRC where decreased* FURIN* expression levels do not influence relapse-free survival, we previously demonstrated in a mouse model for pleomorphic adenomas of the salivary glands that even monoallelic deletion of* Furin* resulted in a significant delay in the tumor formation [2]. Thus, the benefit of decreased FURIN levels depends on the particular tumor type. In hepatocellular carcinoma patients, high* FURIN* expression even predicts a better postoperative disease-free survival [31]. In line with this result,* FURIN* overexpression in hepatocellular carcinoma cell lines significantly suppressed the tumor growth in subcutaneous xenograft experiments compared to the mock control. Thus, depending on the cancer type FURIN inhibition is either beneficial (salivary gland tumors [2], skin cancer [3]) or disadvantageous (hepatocellular carcinoma [31]) or has no clear effect (CRC) on the tumorigenic process.

## 5. Conclusions

In this report, we demonstrate that a SNP in the P1 promoter of* FURIN* does not influence its expression levels in CRC and has no impact on the postoperative disease-free survival and overall survival. Furthermore,* FURIN* expression levels have no impact on the postoperative disease-free survival in CRC. This is in contrast to other tumor types either where* FURIN* expression predicts a better postoperative disease-free survival such as in hepatocellular carcinoma or where FURIN inhibition can delay the tumorigenic process (salivary gland tumors, skin cancer). This demonstrates that the role of FURIN in tumorigenesis depends on the particular tumor type and the affected signaling pathways.

## Figures and Tables

**Table 1:** Genotype distribution of SNP rs4932178 (C-229-T) in the promoter of furin among patients with CRC.

Genotype	Number of patients (%)
CC	**529 (38.7%)**
CT	**650 (47.6%)**
TT	**187 (13.7%)**

Total	**1366 (100%)**

**Table 2:** Cox regression analysis with continuous furin expression values showed no association between furin expression and relapse-free survival for three different probe sets.

Probe set	HR	CI	*P* value
CB852900_s	1.02	0.87–1.20	0.806
NM_002569	0.98	0.77–1.25	0.901
NM_002569_x	0.94	0.72–1.22	0.649

**Table 3:** Distribution of genotypes across clinical and molecular subgroups.

Characteristic	CC (*n* = 502): *n* (%)	CT (*n* = 613): *n* (%)	TT (*n* = 179): *n* (%)	*P*
Stage				
2	172 (34.3%)	177 (28.9%)	52 (29.1%)	0.13144
3	330 (65.7%)	436 (71.1%)	127 (70.9%)	
nstage				
N0	172 (34.3%)	177 (28.9%)	52 (29.1%)	0.17465
N1	226 (45.0%)	276 (45.0%)	82 (45.8%)	
N2	104 (20.7%)	160 (26.1%)	45 (25.1%)	
tstage				
T12	27 (5.4%)	33 (5.4%)	14 (7.8%)	0.5306
T3	385 (76.7%)	480 (78.3%)	130 (72.6%)	
T4	90 (17.9%)	100 (16.3%)	35 (19.6%)	
Grade				
G-12	444 (89.3%)	556 (91.1%)	165 (92.7%)	0.37509
G-34	53 (10.7%)	54 (8.9%)	13 (7.3%)	
Tumor site				
Left	298 (59.4%)	370 (60.4%)	109 (60.9%)	0.92295
Right	204 (40.6%)	243 (39.6%)	70 (39.1%)	
MSI				
MSS	407 (85.0%)	494 (85.5%)	137 (82.0%)	0.54428
MSI-H	72 (15.0%)	84 (14.5%)	30 (18.0%)	
BRAF				
wt	458 (92.5%)	563 (93.1%)	156 (88.6%)	0.15503
mut	37 (7.5%)	42 (6.9%)	20 (11.4%)	
loh18q.2inf				
No LOH	111 (34.8%)	120 (30.7%)	30 (28.6%)	0.37806
LOH	208 (65.2%)	271 (69.3%)	75 (71.4%)	
TS				
75	153 (33.6%)	192 (34.3%)	55 (33.3%)	0.96312
25/50	302 (66.4%)	367 (65.7%)	110 (66.7%)	
SMAD4				
No loss	400 (81.5%)	469 (77.3%)	140 (78.7%)	0.23089
Any loss	91 (18.5%)	138 (22.7%)	38 (21.3%)	
KRAS				
wt	303 (61.2%)	358 (59.5%)	111 (64.5%)	0.47866
mut	192 (38.8%)	244 (40.5%)	61 (35.5%)	
